# Estradiol protects hair cells from cisplatin-induced ototoxicity via Nrf2 activation

**DOI:** 10.1080/13510002.2022.2161224

**Published:** 2023-01-20

**Authors:** Masahiro Adachi, Kota Yanagizono, Yasuhito Okano, Hitoshi Koizumi, Isao Uemaetomari, Keiji Tabuchi

**Affiliations:** a Department of Otolaryngology-Head and Neck Surgery, University of Tsukuba, Tsukuba, Ibaraki, Japan; b Department of Otolaryngology, Graduate School of Comprehensive Human Sciences, University of Tsukuba, Tsukuba, Japan

**Keywords:** Cisplatin, cochlea hair cell, estradiol, GSTA4, Nrf2, organ of corti, ototoxicity, reactive oxygen species

## Abstract

Cisplatin-induced ototoxicity is caused by reactive oxygen species. It has been recognized that estradiol (E2) regulates redox balance. However, little is known about the protective mechanisms of E2 against cisplatin-induced ototoxicity. In this study, we investigated the effect of E2 on nuclear factor erythroid 2-related factor 2 (Nrf2)-mediated hair cell protection using the organ of Corti isolated from mice. The organ of Corti collected from C57BL/6 mice at 3–5 postnatal days was used in all experiments. The organ of Corti was exposed to 20 μM cisplatin with/without 100 nM E2 to examine the effect of E2 on cisplatin-induced hair cell loss. The mRNA expression of Nrf2 and the phase II detoxification gene after E2 and cisplatin treatment was analyzed using quantitative real-time PCR. E2 significantly reduces cisplatin-induced cochlear hair cell death. In addition, 100 nM E2 increased the mRNA expression of Nrf2 and phase II detoxification genes in the organ of Corti under cisplatin treatment. Our results suggest that E2 activates Nrf2, phase II detoxification enzymes and exerts a protective effect against cisplatin-induced ototoxicity.

## 1. Introduction

Cisplatin-based chemotherapy is the mainstay of cancer treatment [1]. However, hearing loss is a serious and permanent side effect of cisplatin-based chemotherapy [2]. Over 40% of the patients who receive cisplatin administration are left with permanent hearing loss [1]. Cisplatin-induced ototoxicity is associated with the loss of cochlear hair cells, mainly outer hair cells [3,4]. The main cause of cisplatin-induced ototoxicity is the generation of reactive oxygen species (ROS) [5].

Nuclear factor erythroid 2-related factor 2 (Nrf2) has been found to be involved in the responses to oxidative stress [6,7]. Nrf2 regulates the transcription of genes encoding phase II detoxification enzymes through antioxidant response elements (ARE) [6]. Under normal conditions, Kelch-like ECH-associated protein 1 (Keap1) inhibits the interaction between Nrf2 and ARE [8]. However, under oxidative stress, such as ROS, Keap1 changes its structure, and Nrf2 accumulates in the nucleus and activates ARE-mediated gene transcription [6]. Previous reports have shown that the Nrf2-ARE pathway is involved in the antioxidant activity against cochlear hair cell loss [3,7,9–11]. Zhang et al. reported that Nrf2 activation protects auditory hair cells by suppressing the total cellular ROS levels [7].

Estrogen is a major female hormone that promotes the development and maturation of female reproductive functions [12]. Natural estrogens consist of four distinct forms. Estradiol (E2) is the most potent and abundant form of estrogen [12]. Previous reports have indicated that E2 protects skeletal muscles, central neurons, and the cardiovascular system by regulating redox balance [12,13]. The protective role of E2 in the auditory system has also been reported [14,15]. However, little is known about the protective mechanisms of E2 against cisplatin-induced ototoxicity. In this study, we investigated the effect of E2 on the Nrf2-mediated hair cell protection mechanism using the organ of Corti isolated from C57BL/6 mice.

## 2. Materials and methods

### 2.1. Animals and cochlear explants

C57BL/6 mice at 3–5 postnatal days were used in this study. For cochlear explant culture, we used the dissected organ of Corti based on the method of Van de Water and Ruben and Sobkowicz et al [16–18]. All the mice were treated in accordance with the guidelines of the Laboratory Animal Research Center of the University of Tsukuba.

### 2.2. Cisplatin treatment

Cochlear explants were cultured in Dulbecco’s modified Eagle’s medium (DMEM) supplemented with 10% fetal bovine serum (FBS), 25 mM HEPES, and 30 U/ml penicillin. The cells were incubated at 37 °C with 5% CO_2_ and 95% humidity. Cisplatin cultures were maintained in medium overnight (8–12 h) and then exposed to 5, 10 or 20 µM cisplatin for 48 h to examine hair cell loss. Cisplatin (Randa®, Nippon Kayaku, Tokyo Japan) was initially dissolved in saline and then diluted to the final concentration immediately before use [19].

### 2.3. E2 treatment

E2 was initially dissolved in ethanol to 10 mM and then diluted to the final concentration immediately before use [14]. The organ of Corti explant was stabilized in the culture medium overnight (8–12 h), and each group was exposed to a culture medium containing 20 µM cisplatin and 100 nM E2 for 48 h to examine cisplatin-induced hair cell loss under E2 treatment.

### 2.4. Cytochemistry

The cultured explants were fixed for 20 min with 4% paraformaldehyde in PBS and permeabilized with 5% Triton^TM^ X-100 (Sigma, St. Louis, MO) in PBS with 10% FBS for 10 min. The specimens were stained with a rhodamine-phalloidin probe (1:100; Invitrogen, Carlsbad, CA) at 15-25°C for 1 h. Phalloidin binds F-actin with nanomolar affinity and labels stereociliary arrays and cuticular plates of hair cells. The specimens were observed under a fluorescence microscope (BZ-X710; Keyence, Osaka, Japan). If no stereocilia or cuticular plate were observed with phalloidin staining, the hair cells were considered missing. Quantitative results were obtained by evaluating 30 outer hair cells associated with 10 inner hair cells [20]. The average of three separate counts was representative of each culture. The residue of hair cells was measured as a percentage.

### 2.5. Quantitative real-time PCR (qPCR) analysis

The organ of Corti was stabilized in the culture medium overnight and exposed to the medium under different conditions. To analyze the effect of E2, explants were exposed to medium containing 10 or 100 nM E2 for 8 and 24 h. To analyze the effect of E2 on cisplatin conditions, plants were exposed to 20 μM cisplatin or 20 μM cisplatin medium containing 100 nM E2 for 8 and 24 h. The organ of Corti was homogenized in ISOGEN (Nippon Gene, Tokyo, Japan), and total RNA was purified according to the manufacturer’s instructions for each sample. Reverse transcription was performed using a Veriti Thermal Cycler (Applied Biosystems, Waltham, MA), and cDNA was synthesized using ReverTra Ace Reverse Transcriptase (Toyobo, Osaka, Japan). qPCR was performed in QuantStudio5 (Applied Biosystems, Waltham, MA) using Thunderbird SYBR qPCR mix (Toyobo, Osaka, Japan) with a SYBR green system. Primers used in this study are shown in Table 1. β-Actin was used as an internal control.

**Table 1.** Primer sequence for quantitative real-time PCR.

Gene	Forward primer (5′ – – 3′)	Reverse primer (5′ – – 3′)	Accession number
Mouse Nrf2	CAAGACTTGGGCCACTTAAAAGAC	AGTAAGGCTTTCCATCCTCATCAC	NM_010902.5
Mouse Beta-Actin	CGGTTCCGATGCCCTGAGGCTCTT	CGTCACACTTCATGATGGAATTGA	NM_007393.5
Mouse Gclc	ATCTGCAAAGGCGGCAAC	ACTCCTCTGCAGCTGGCTC	NM_010295.2
Mouse Sod2	GACAAACCTGAGCCCTAAG	CGACCTTGCTCCTTATTG	NM_013671.3
Mouse Gsta4	GGGAACAGTATGAGAAGAAGATGCAAAA	CCCATCGATTTCAACCAAGG	NM_010357.3
Mouse Nqo1	AGCTGGAAGCTGCAGACCTG	CCTTTCAGAATGGCTGGCA	NM_008706.5

### 2.6. Data analysis

All data are presented as mean ± SD. Student’s t-test and one-way analysis of variance (ANOVA) with Tukey’s post-hoc test were performed. All statistical analyses were performed using EZR (Saitama Medical Center, Jichi Medical University, Saitama, Japan), which is a graphical user interface for R (The R Foundation for Statistical Computing, Vienna, Austria) [21]. Statistical significance was set at *p* < 0.05.

## 3. Results

### 3.1. Cochlear hair cell count

**Figure 1.**
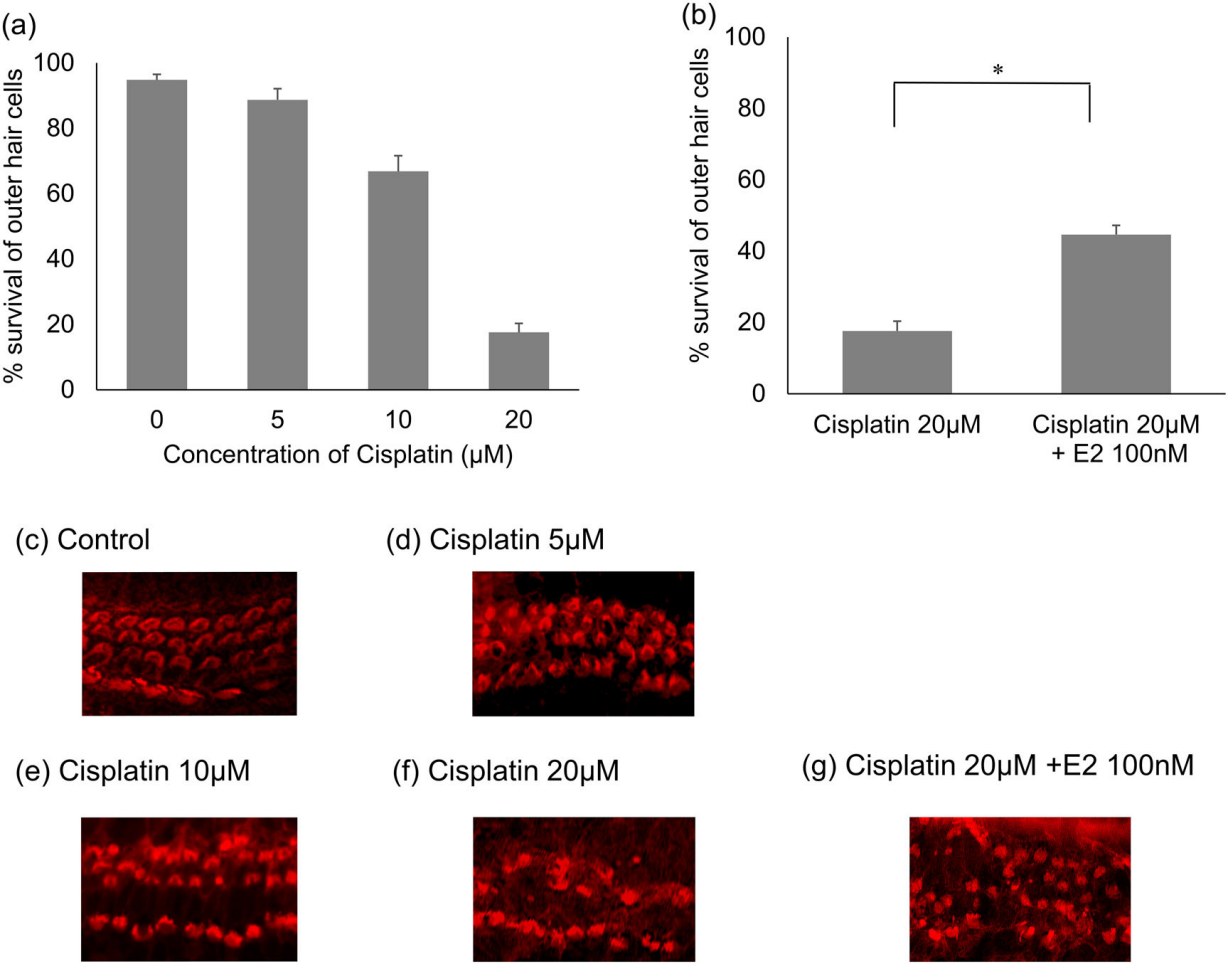
Cochlear outer hair cell survival in cisplatin ototoxicity. (a) Quantitative analysis of cochlear outer hair cell of each concentration of cisplatin. *n* = 6, Error bars represent ± s.e.m. (b) Effect of Estradiol on cochlear outer hair cell survival in cisplatin ototoxicity. *n* = 6, *p < 0.001 (Student t test). Error bars represent ± s.e.m. (c-g) Representable images of cochlear hair cells exposed to cisplatin; (c) control; (d) 5 µM of cisplatin; (e) cisplatin 10 µM; (f) cisplatin 20 µM: (g) cisplatin 20 µM and E2 100 nM. E2: Estradiol.

The effects of cisplatin concentration on the outer hair cells were examined (Figure 1a). The explants treated exclusively with cisplatin showed significantly reduced numbers of outer hair cells. Based on these results, the cisplatin concentration used in this study was chosen to be 20 µM. Figure 1b shows the effects of E2 on cisplatin-induced cochlear hair cell loss. Compared to explants treated with 20 µM cisplatin alone, explants treated with 20 µM cisplatin and 100 nM E2 had a significantly higher rate of residual outer hair cells.

### 3.2. The effect of E2 on Nrf2 expression

**Figure 2.**
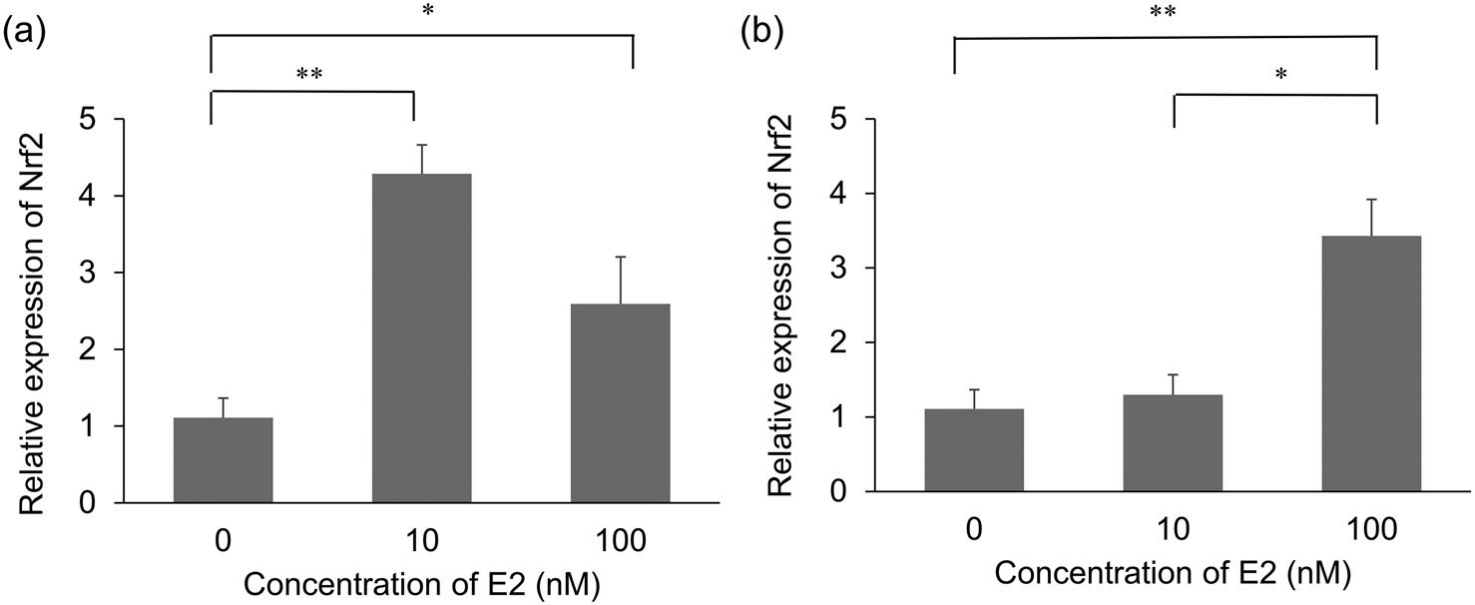
Assessment of mRNA expression levels of Nrf2 after Estradiol (E2) treatment. (a) mRNA expression levels of Nrf2 after 8 h of E2 treatment. *n* = 4, Error bars represent ± s.e.m. (b) mRNA expression levels of Nrf2 after 24 h of E2 treatment. *n* = 4, Error bars represent ± s.e.m. E2: Estradiol. For all experiments, *p < 0.05 **p < 0.001.

The expression of Nrf2 mRNA in the organ of Corti was analyzed using qPCR post 8 and 24 h E2 treatment. (Figure 2) The explants treated with 100 nM E2 showed significantly higher Nrf2 mRNA expression than the control explants at both 8 and 24 h. In contrast, explants treated with 10 nM E2 showed significantly higher Nrf2 mRNA expression than control explants only at 8 h.

### 3.3. The effect of E2 on Nrf2 expression under cisplatin treatment

**Figure 3.**
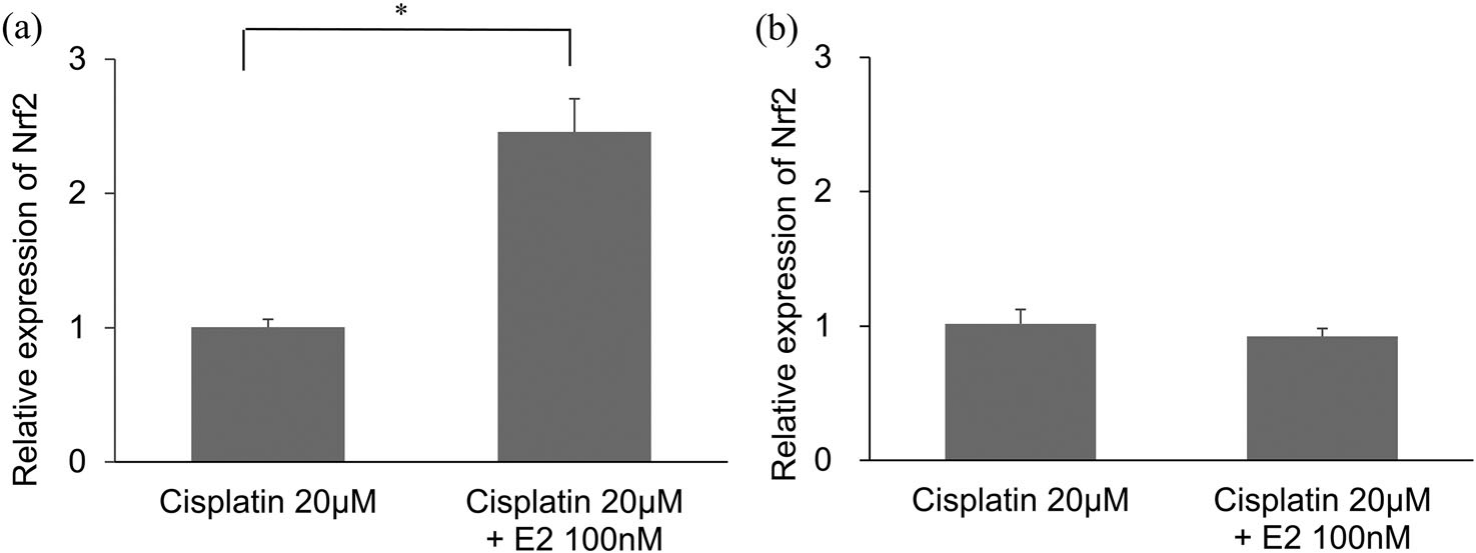
Assessment of mRNA expression levels of Nrf2 after Cisplatin and Estradiol (E2) treatment. (a) mRNA expression levels of Nrf2 after 8 h post Cisplatin and E2 treatment. n = 4, Error bars represent ± s.e.m. (b) mRNA expression levels of Nrf2 after 24 h post Cisplatin and E2 treatment. n = 4, Error bars represent ± s.e.m. E2: Estradiol. For all experiments, *p < 0.001.

**Figure 4.**
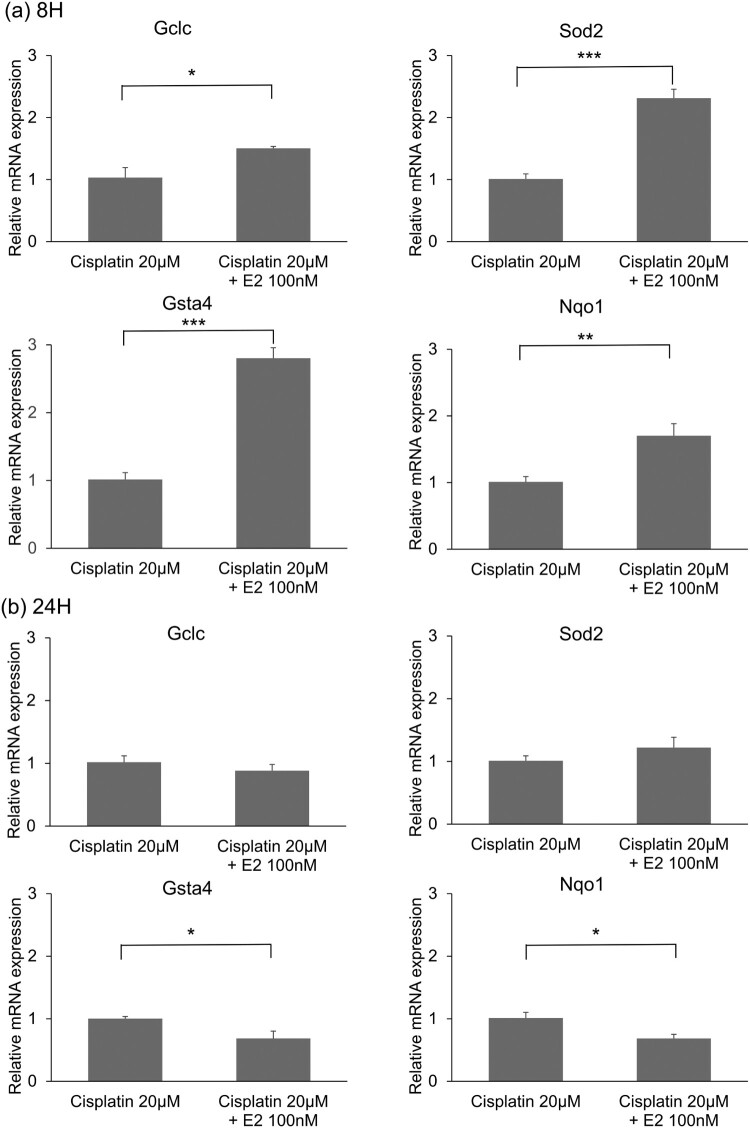
Assessment of mRNA expression levels of detoxification genes after Estradiol (E2) treatment. (a) mRNA expression levels of detoxification genes after 8 h of E2 treatment. n = 4, Error bars represent ± s.e.m. (b) mRNA expression levels of detoxification genes after 24 h of E2 treatment. n = 4, Error bars represent ± s.e.m. E2: Estradiol. For all experiments, *p < 0.05, **p < 0.01, ***p < 0.001.

The expression of Nrf2 mRNA in the organ of Corti post 8 h of treatment with Cisplatin and E2 was significantly higher than that in the organ of Corti treated with cisplatin exclusively (Figure 3a). When measured at 24 h, there were no statistical differences in Nrf2 expression between cisplatin combined E2 treatment and cisplatin treatment alone (Figure 3b). The analysis of detoxification genes showed that the expression of *Gclc, Nqo1, SOD2, and Gsta4* was significantly increased at 8 h (Figure 4).

## 4. Discussion

In this study, we found that E2 had a protective effect against cisplatin-induced hair cell loss. The qPCR results indicated that E2 activated Nrf2 and the transcription of genes encoding phase II detoxification enzymes. This suggests that E2 reduces cisplatin-induced ototoxicity by activating Nrf2.

Many drugs have been shown to protect hair cells [3,5,7,9,10,18,22,23]. Previous reports have indicated that Nrf2 has been shown to be a critically important regulator for protecting hair cells from cisplatin ototoxicity [3,7,9,10,23]. Zhang et al. showed that Nrf2 activation protects auditory hair cells from cisplatin ototoxicity in HEI-OC1 cells [7]. In addition to previous studies using HEI-OC1 cells, Hoshino et al. demonstrated the protective effect of Nrf2 in the organ of Corti dissected from mice [6].

Gender differences in cisplatin ototoxicity have been reported [12,24]. Sex hormones, particularly estrogen, are thought to have protective effects against hair cell loss in female patients [24]. However, the mechanism by which E2 protects against cisplatin-induced hair cell loss is not well understood. Hu et al. reported that estrogen-deficient ovariectomized rats had significantly decreased DPOAE and increased ABR threshold under cisplatin treatment [15]. Park et al. reported that GSTA4, a member of the phase II detoxifying enzyme superfamily, induces detoxification and plays an important role in estrogen-mediated neuroprotection [4]. In this study, we indicated that E2 itself has a protective effect against cisplatin ototoxicity and that this effect is activated by Nrf2 and phase II enzymes, including GSTA4.

In our study, a significant increase in the mRNA levels of Nrf2 and phase II detoxification enzymes was observed after 8 h of administration of cisplatin with E2 compared with cisplatin alone. However, at 24 h after drug exposure, there was no significant difference in the mRNA expression of Nrf2 and its related detoxification enzymes between the E2-treated and non-treated groups. The activation of Nrf2 is a rapid cellular response that increases immediately after cisplatin treatment [13]. Regarding the time of Nrf2 expression, Jo et al. showed that Nrf2 increases up to 8 h, then decreases, and after 24 h, it is lower than the control levels in HEI-OC1 cells [10]. Their result conforms to our results and suggests that the expression of Nrf2 is time-dependent.

This study had several limitations. First, the protein expression of Nrf2 and related detoxifying agents was not assessed. This was due to the considerably small size of organs of Corti from post-neonatal mice (3-5 days old), making it difficult to measure the protein expression. In previous studies using cell lines derived from the mouse organ of Corti, an association between mRNA and protein levels has been reported for Nrf2 and Phase II detoxification enzymes [3,7]. We believe that this study offers valuable insights into the Nrf2-mediated protective effect of E2. Second, the concentration of E2 exceeded the safe serum E2 level [14]. For example, Hellgren et al. showed that the serum E2 level of women during E2 replacement treatment was 60–90 pM [14,25]. However, our results showed a probable otoprotective mechanism of E2, which is not well known.

In conclusion, E2 activates Nrf2, phase II detoxification enzymes and exerts a protective effect against cisplatin-induced ototoxicity. Our results provide an insight to comprehend the sex-based differences in cochlear function. Thus, E2 may be a possible candidate for the protection of hair cells against cisplatin.

## Data Availability

Data used to support the findings of this study are available from the corresponding author upon request.

## Submission declaration

We declare that its publication has been approved by all the authors.
